# Conceptualizing myocardial contractility as an emergent property that characterizes myocardial contraction

**DOI:** 10.3389/fphys.2025.1499536

**Published:** 2025-04-16

**Authors:** Serena Y. Kuang, Gorune Geloian

**Affiliations:** Department of Foundational Medical Studies, Oakland University William Beaumont School of Medicine, Rochester, MI, United States

**Keywords:** myocardial contractility, complex adaptive system, emergent property, nonlinearity, Ca^2+^ transient, capacity, adaptability, circadian rhythm, system-level property

## Abstract

Myocardial contractility (MC) is a fundamental concept that is widely used to describe the cardiac muscles’ mechanical function, yet its definitions in textbooks and literature are vague, inconsistent, and often contradictory. In this article, we categorize these many issues into five groups and conducts a conceptual analysis to redefine MC from a broader, more comprehensive perspective. We propose a functional, three-domain framework of MC consisting of capacity/resource, adaptability, and ability (force (F) and/or velocity (V) generated during muscle contraction), emphasizing the dynamic, non-linear interactions among the three domains and their clinical significance. Specifically, we highlight how interventions targeting MC may produce non-linear effects, suggesting a shift toward optimizing resource use rather than maximizing outputs (i.e., F and/or V of myocardial contraction, the outputs of the ability domain), which could potentially reduce the complications of positive inotropic interventions. We also discuss the implications of several new conceptual developments as the byproducts of the three-domain MC framework. Additionally, we identify system-level emergent properties of MC briefly, including contraction efficiency, circadian rhythm-dependence, temperature-dependence, and history-dependence, with implications for cardiac muscle research, exercise training, and clinical decision-making. The three-domain functional framework of MC resolves the inconsistencies in definitions, differentiates MC from cardiac performance, and offers a structured perspective for facilitating both experimental studies and therapeutic strategies.

## 1 Introduction

This introduction includes background information, definitions of key terms, and the positioning of myocardial contractility (MC) within the heart.

### 1.1 Background

In a *Frontiers* article titled “Myocardial Contractility: Historical and Contemporary Considerations,” (Muir and Hamlin, 2020, p1) noted,

“The term myocardial contractility is thought to have originated more than 125 years ago and has remained an enigma ever since. Although the term is frequently used in textbooks, editorials and contemporary manuscripts, its definition remains elusive, often being conflated with cardiac performance or inotropy. The absence of a universally accepted definition has led to confusion, disagreement and misconceptions among physiologists, cardiologists and safety pharmacologists.”

They recommended “a modern definition of myocardial contractility as the preload, afterload and length-independent intrinsic kinetically controlled, chemo-mechanical processes responsible for the development of force and velocity” (p1). This definition is insightful but vague. Facing the many issues in the definition of MC, we previously proposed understanding it comprehensively through three dimensions: capacity, adaptability, and ability (Geloian and Kuang, 2023). Since the term “three dimensions” could mislead readers into perceiving MC as a 3-D structural concept, this article will instead adopt the term “three functional domains of MC.” In this framework, capacity, adaptability, and ability are no longer described as three dimensions of MC but rather as three interwoven functional domains, each contributing uniquely to the dynamic behavior of myocardial contraction. In response to Muir and Hamlin (2020) and to further develop our three-domain conceptualization of MC, we apply the concept of complex adaptive system (CAS) to advance the understanding of MC by the following steps.

First, we comprehensively address and analyze the many issues with the existing definitions and classify the issues into five groups. Through this conceptual analysis process, why the definition of MC should include the three domains becomes clear.

Second, we address the rationales to conceptualize MC from a broader scope, further develop the three-domain conceptualization of MC into a dynamic open framework that illustrates the internal functional organization of MC, and explain how the framework improves diagnostic and therapeutic strategies. This framework eliminates all the issues we address and puts the three domains and their components in place; moreover, it elaborates on the rich connotations of Muir and Hamlin’s modern definition, making it clear and complete.

Third, we present the theoretical and practical significance/implications of several new conceptual developments in this article.

Finally, considering the complex MC or the framework as a system, we briefly highlight its system-level properties that apply to all three domains and their implications.

This article advances the understanding of MC, promotes the CAS way of thinking to study complex biological systems, and is of both conceptual and practical value in research on cardiac muscle contraction, exercise training, and clinical practice.

### 1.2 Definitions of key terms

A complex adaptive system (CAS) consists of networks of interacting components, or agents, that display emergent properties and resilience (Holland, 2006; Carmichael and Hadžikadić, 2019). These systems can adapt to internal and external perturbations. All biological systems, including cells, tissues, organs, and entire organisms, are CASs (Janson, 2012). The heart functions as a cardiac CAS, comprising right and left atrial CASs and right and left ventricular CASs, with cardiomyocytes (cellular CASs) as agents.

An emergent property is a characteristic or behavior of a CAS that arises from the complex interactions of its components but often cannot be predicted by examining the components in isolation. Emergent properties are central to understanding a CAS as they emphasize how collective behavior results from multi-level interactions rather than isolated components. Examples are provided in Section 1.3 below.

Nonlinear dynamics, or nonlinearity, is an important property of a CAS. The system’s response to internal or external perturbations is often unpredictable from the properties of the components alone; it may respond proportionately or disproportionately or have no response (Ladyman et al., 2013).

Inotropy refers solely to the force (F) of muscle contraction (Muir and Hamlin, 2020).

Clinotropy refers solely to the velocity (V) of muscle contraction (Muir and Hamlin, 2020).

Ca^2+^ modulators are extrinsic factors that increase or decrease Ca^2+^ transient levels in the cytosol during muscle contraction, including sympathetic tone, cardiac glycosides, and β-blockers. These extrinsic factors are typically termed “inotropic factors.” They also affect the speed and duration of Ca^2+^ transient, influencing both force (F) and velocity (V), making “Ca^2+^ modulators” a more accurate term. An increase in sympathetic tone and/or an administration of cardiac glycosides are positive Ca^2+^ modulators, and β-blockers are negative Ca^2+^ modulators.

Preload varies in definition based on context: for the whole heart, it often refers to end-diastolic volume (EDV) or pressure (EDP), while for myocardial tissue, it involves the initial fiber length or stretch. *In vitro*, preload can be seen as the load stretching the fiber, though it is often approximated using initial fiber length, resting tension, or sarcomere length. As this article focuses on myocardial tissue, these proxies are appropriate for preload measurement.

Afterload is the resistance the heart must overcome to eject blood during systole, mainly determined by aortic pressure, vascular resistance, and aortic valve properties. At the myocardial level, it reflects the force myocardial fibers generate to shorten against an opposing load after contraction begins. Afterload can be better understood by examining the mechanics of muscle contraction and how muscle fibers respond to varying external loads:

• Isometric contraction: Muscle develops tension without shortening, as the load exceeds its ability to contract.
• Isotonic contraction: Muscle changes length under constant tension, shortening (concentric) or lengthening (eccentric) while moving a load.

3-imEFs refers to the collective grouping of three types of immediate, extrinsic factors that modulate the production of F and/or V: preload, Ca^2+^ modulator, Afterload. Why these three types of extrinsic factors are grouped as 3-imEFs will become clear later.

### 1.3 Positioning of MC within the heart

Heart rate and cardiac oxygen consumption rate are emergent properties of the entire heart. Cardiac performance may refer to the performance of the entire heart, but it more frequently refers to the performance of the left ventricle. End diastolic volume, end systolic volume, peak systolic ventricular pressure, stroke volume, ejection fraction, cardiac output, pressure-volume loop, and end systolic pressure-volume relationship (ESPVR) are emergent properties that apply to the left ventricle. They describe cardiac performance from different aspects.

Myocardial tissue has both passive and active emergent properties. Passive properties, like structural integrity and viscoelasticity, require minimal energy. Active properties are self-reinforcing processes (e.g., automaticity, excitability, and conductivity) or significant energy-dependent processes (e.g., Ca^2+^ handling, ATP production, and muscle contraction). Muscle contraction, an active property, involves contraction, relaxation, and fatigue resistance, emerging at the myocardial tissue level (multiple cardiomyocytes) rather than the whole heart. MC reflects the collective contractility of cardiomyocytes, which share structural and functional similarities but also exhibit adaptive heterogeneities (Michel et al., 2022) that influence tissue-level MC. Thus, MC is not a simple algebraic summation of individual cellular contractility.

Heart or left ventricular-level parameters like stroke volume, ejection fraction, P-V loop, and ESPVR are emergent properties of the heart that may not directly reflect MC at the tissue level. For example, if some cardiomyocytes lose contractility due to ischemia but adjacent cells compensate, normal cardiac output may persist, illustrating the nonlinear response of the cardiac CAS to internal perturbations.

## 2 Outlining the three domains of MC by addressing and analyzing the major issues in the definitions of MC

**FIGURE 1:**
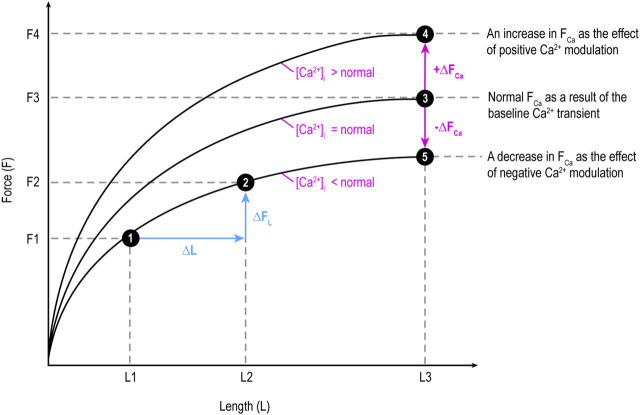
Illustration of the two components of force (F): length-dependent component (F_L_) influenced by preload and Ca^2+^-dependent component (F_Ca_) determined by Ca^2+^ transient level and modulated by Ca^2+^ modulators. ∆F_L_: a change (increase or decrease) in F_L_; ∆F_Ca_: a change in F_Ca_. F changes along a curve due to a change in F_L_, while shifts between curves at the same X-axis length (e.g., from 3 to 4 or 3 to 5) is due to a change in F_Ca_. This figure applies the original Frank-Starling law describing heart-level behavior to myocardial tissue.

This section classifies the issues into five groups and analyzes them, explaining why MC should be understood through three domains: capacity, adaptability, and ability. Figure 1 illustrates the components of F and influencing factors, serving as a standard for defining and analyzing MC.

### 2.1 There is no agreement about how MC should be defined

This issue is reflected in the following descriptions:

• MC is “a somewhat vague but clinically useful term that distinguishes a better-performing heart from a poorly performing one” (Boulpaep, 2017, p528).
• After a comprehensive study of the various measures of MC, Blinks and Jewell (1972) concluded that “it seems unlikely that any simple index of contractility can be expected to be universally applicable” (p254) and “It is not possible to give a rigorous definition of ‘myocardial contractility’ as such. One can only describe the contractile state of the heart, and it is best done in terms of the relations among three prime variables—force, length, and time—and a derived variable—velocity” (p258).
• “Finally, note that the term ‘contractility’ is used to define the ability of heart muscle to develop force at a fixed length (or pressure at fixed ventricular volume). However, a variety of correlative physiological parameters based on the properties of muscle (i.e., rate of rise of force, maximal velocity of shortening) are also used to define contractility and are hotly debated” (Sweeney and Hammers, 2018, p9).
• “Ejection fraction is the most commonly used clinical index to assess contractility. The end-systolic volume-pressure relationship is the most accurate index available” (Downey, 2003a, p202).
• “The definition of myocardial contractility remains complex and its exact measurement remains difficult, especially in the intact heart” (Opie, 1995, pS1).
• “In view of the vagueness of the definitions, it may be worthwhile in the future to omit this term (i.e., contractility) entirely from the literature on cardiac mechanics” (Mirsky, 1974, p7).

These descriptions at least reflect that MC is not simple but complex.

### 2.2 Contractility and inotropy are considered the same thing and used interchangeably

This situation is very common (Muir and Hamlin, 2020). A few examples are given below.

• “There are numerous techniques which attempt to quantify inotropy (or myocardial contractility)” (Smith and Madigan, 2013, p580).
• “The force generated by cardiac muscle cells depends on the cells’ contractility, sometimes called the inotropic state” (Downey, 2003b, p183).
• “It is important for both physiologists and clinicians to distinguish alterations in the performance of the heart resulting from changes in inotropic state from those changes caused by alterations in loading conditions. In this text, the term ‘contractility’ will be used to refer to the inotropic state of the heart” (Rhoades and Bell, 2018, p893).

It will become clear that contractility encompasses much more than inotropy.

### 2.3 The clinical and academic understandings of inotropy are inconsistent

Etymologically speaking, inotropy means F (Muir and Hamlin, 2020). It does not encompass V (clinotropy). F generated by myocardial tissue has two components: length-dependent (F_L_) and Ca^2+^-dependent (F_Ca_; Pieske et al., 1998), as shown in Figure 1. Hence, etymologically, logically, and conceptually, inotropy as F encompasses both F_L_ and F_Ca_. However, it is quite common that only F_Ca_ is considered inotropy:

• “A modification of the contractile ability of muscle at the cellular level, independent of loading conditions, is said to be a modification of the inotropic state or contractility of the muscle” (Rhoades and Bell, 2018, p893).
• “When heart muscle contracts, all muscle fibers are activated and the only mechanisms that can alter force generation are changes in fiber length (preload; length-dependent activation) and changes in inotropy (length-independent activation)” (Klabunde, 2023, para. 1).
• “Inotropy is the strengthening/weakening function of the cardiac tissue: Positive = calcium release causes strengthening; Negative = hypercalcemia or weakening” (Al-Shura, 2014, para. 1).

In practical application, many inotropic agents are drugs that influence the level of Ca^2+^ transient such as β-agonists, β-blockers, and cardiac glycosides; many inotropic therapies are intended to increase F_Ca_ (a result of an increase in Ca^2+^ transient) (Marks, 2013; Yang et al., 2023; Arfaras-Melainis et al., 2023). Cardiac physiologists, pharmacologists, clinicians, and others should discuss this issue to reach a consensus. If inotropy encompasses both F_L_ and F_Ca_, then inotropic factors or agents should be changed to Ca^2+^ modulators as we do in this article. If inotropy refers to F_Ca_ only, then we should redefine inotropy as F_Ca_.

### 2.4 MC is considered the “ability” of the heart, but this ability is defined inconsistently

Muir and Hamlin (2020) noted that some consider MC the intrinsic ability of the heart to generate F and shortening independent of preload and afterload at a fixed heart rate, while others consider V_max_ against zero afterload to be equal to the complete measure of it, and still others consider contractility not only the ability of the heart to develop F but also the ability to generate V. Muir and Hamlin (2020) agree with the third option, but it is unclear what “ability” is and whether it is dependent on or independent of extrinsic factors (preload, afterload, heart rate, etc.). In addition, ventricular-level properties are used to describe tissue-level contractility, which is fine if all myocytes are healthy, but if a fraction of myocytes do not function well, using the ventricular-level properties to describe contractility at the myocardial tissue level is inappropriate.

In our opinion, F and V represent different manifestations of myocardial tissue’s ability, and as we will explore later, the complexity of contractility cannot be reduced to a simple ability. This is why we propose that “ability” is just one domain of MC (Geloian and Kuang, 2023) and should be differentiated from the higher-level ability and properties of the heart (e.g., pumping, pressure, stroke volume, ejection fraction, etc.).

### 2.5 Considering myocardial tissue’s ability to be intrinsic and independent of extrinsic factors is self-contradictory

MC is commonly considered the heart muscle’s *intrinsic contractile property* or *intrinsic ability* to generate force and shorten; the intrinsic property is considered *independent of* the extrinsic preload and afterload (Muir and Hamlin, 2020). This view is self-contradictory because myocardial tissue’s ability (F and/or V) is highly extrinsic factor-dependent (addressed below); in terms of MC, what factors can be considered intrinsic have not yet been explicitly identified, nor has whether intrinsic factors can be independent of extrinsic factors. We address these issues below.

#### 2.5.1 Myocardial tissue’s ability is highly dependent on the 3-imEFs

The 3-imEFs-dependency of myocardial tissue’s ability is well-known as follows:

Preload-dependency of the ability domain:

• As preload increases (stretching the muscle fiber), the muscle can generate greater isometric force (P_0_) up to an optimal length, beyond which force generation decreases (length-tension (L-T) relationship).
• Generally, an increase in preload (up to the optimal length) can lead to a higher V_max_ due to more optimal overlap of actin and myosin filaments, which facilitates cross-bridge cycling (Allen and Kentish, 1985).

Ca^2+^ modulator-dependency of the ability domain:

• The stronger the positive Ca^2^⁺ modulation, the higher the level of Ca^2^⁺ transient, the greater the F and V.
• The opposite is true for negative Ca^2^⁺ modulation.

Afterload-dependency of the ability domain:

• If a given afterload is immoveable, then the myocardial contraction is isometric, and the muscle’s ability is expressed as P_0_, a pure manifestation of F. Under this circumstance, P_0_ equals the complete measure of the myocardial ability, not the complex contractility.
• If the afterload is zero, then the ability is expressed as V_max_, a pure manifestation of V. Under this circumstance, V_max_ against zero afterload equals the complete measure of the myocardial ability, not the complex contractility.
• If the afterload is non-zero and can be moved, then the myocardial contraction is isotonic and the muscle’s ability is expressed through both F and V.

The greater the given afterload, the greater the F, but the slower the V, and *vice versa*. Since the F-V relationship is derived from F and V, we consider F (or P_0_) and V (or V_max_) to be the primary manifestations of the muscle’s ability and the F-V relationship the secondary manifestation. Other F- or V-related relationships are also considered secondary manifestations of the muscle’s ability, such as L-T (length-tension), F-L (force-length), time-force, time-velocity, force-frequency, and the power of muscle contraction, which is the product of F and V.

The if-then scenarios outlined above suggest an intrinsic algorithm that determines how the myocardial ability is expressed in response to different afterloads. This intrinsic algorithm demonstrates the acute adaptability of myocardial contraction to the extrinsic afterload. The 3-imEFs-dependency of myocardial ability indicates that myocardial contraction is highly adaptive.

Defining the ability at a fixed heart rate and independent of preload and afterload reflects a tendency to find out something intrinsic and not influenced by extrinsic factors. This effort is not successful, as shown above. Instead, the 3-imEFs-dependent ability is mistaken for intrinsic “contractility.” This tendency also reflects a non-dynamic, rigid way of thinking, leading to a rigid definition of the complex MC, which should be open and dynamic.

Recent research has identified more immediate extrinsic factors that modulate myocardial function by directly interacting with the contractile machinery of the heart muscle. These agents, often referred to as myotropes, include omecamtiv mecarbil and mavacamten. Each exerts distinct effects on the chemomechanical cycle of cardiac myocytes.

Omecamtiv Mecarbil (OM), a myosin activator, enhances MC by directly binding to the myosin heads within the sarcomere. OM increases the number of myosin heads that can interact with actin filaments during systole, thereby prolonging the duration of the force-generating phase of the cross-bridge cycle. This action leads to stronger cardiac contractions without increasing intracellular calcium levels or myocardial oxygen consumption, potentially improving cardiac performance in conditions like systolic heart failure (Malik et al., 2011; Rhoden et al., 2022). In addition, Levosimendan binds to cardiac muscle troponin C, enhancing calcium sensitivity and improving the myocardial ability (Conti et al., 2021).

Mavacamten (Mava) is a selective, allosteric, and reversible inhibitor of cardiac myosin. By stabilizing myosin in its inactive state, Mava reduces the number of myosin-actin cross-bridges formed during the cardiac cycle. This reduction in active myosin heads decreases MC, which is particularly beneficial in conditions characterized by hypercontractility, such as hypertrophic cardiomyopathy. By modulating excessive contractile function, Mava helps alleviate left ventricular outflow tract obstruction and improves diastolic compliance (Spertus et al., 2021; Braunwald et al., 2023).

Myotropes represent a significant advancement in the pharmacological modulation of MC, offering targeted therapeutic options that directly influence the chemomechanical processes of cardiac muscle contraction. Their development underscores the importance of understanding and manipulating the molecular mechanisms of the cardiac sarcomere to treat various cardiac conditions.

#### 2.5.2 What is truly intrinsic in terms of myocardial contraction?

Previously, we proposed that myocardial capacity could be defined as a theoretical, extrinsic-independent force and velocity capacity, with ability (F and/or V) always representing a fraction of it (Geloian and Kuang, 2023). However, this linear approach is inadequate, as no such homogenous capacity exists. Muscle contraction is a non-linear process by which mechanical energy (F and/or V) and heat are both generated. Efficiency of muscle contraction, defined as mechanical energy divided by total energy expended, is relatively low, with cardiac muscle efficiency around 35%–45% (Gibbs and Barclay, 1998). This inefficiency is not a flaw but a result of evolutionary optimization balancing strength, endurance, and energy use (Marino, 2022).

Given the complexity of muscle contraction, capacity cannot be described solely by maximal force, velocity, or energy expenditure. Instead, capacity should be conceptualized as the availability of three intrinsic, genetically determined resources used to generate muscle ability (F and/or V):

Filament Resources: Structured thin and thick filaments provide a fixed number of myosin binding sites and myosin heads (motors). Preload influences the degree of filament overlap, determining the fraction of total myosin heads that can engage in contraction, but it cannot alter the total number of binding sites available. The preload-filament resource interaction allocates motors, making a contraction possible.

Ca^2^⁺ Handling Resources: Ca^2^⁺ handling involves the sarcoplasmic reticulum (SR), Ca^2^⁺ channels, pumps, and buffers (Fearnley et al., 2011). The baseline Ca^2^⁺ transient can be modulated by Ca^2^⁺ modulators, but its maximal and minimal levels are defined by intrinsic resources. These resources set the upper and lower boundaries for Ca^2^⁺ availability and recycling efficiency, which determine the number of myosin binding sites activated. The interaction between Ca^2^⁺ modulators and Ca^2^⁺ handling resources utilizes the allocated motors, making a contraction feasible.

Biochemical Resources: ATP production capacity is determined by the metabolic pathways and enzymes responsible for generating energy for Ca^2^⁺ reuptake and power stroke generation. While ATP production can be modulated by metabolic demands (e.g., exercise), its range remains constrained by intrinsic biochemical resources. The afterload informs the strength of the force and/or the velocity of muscle shortening, which determines the number and rate of ATP hydrolysis. The interaction between afterload and biochemical resources determines the output (F and/or V), making a contraction realizable or achievable.

These three resource types collectively form the capacity domain and are independent of external modulation, though they interact dynamically with extrinsic factors. Each resource type has its own inherent capacity, and the transition from capacity to ability involves non-linear processes. Thus, the capacity domain does not represent maximal force or velocity directly but rather the availability of resources for generating ability. Hence, the capacity domain can also be called the resource domain.

Table 1 summarizes the key factors driving the progression of muscle contraction from possibility to feasibility and ultimately to realizability.

**TABLE 1:** Key intrinsic and extrinsic factors contributing to the progression of a contraction.

	Possibility	Feasibility	Realizability
Type of Resource	Filament resources	Ca^2+^ handling resources	Biochemical resources
Type of Extrinsic Factor	Preload	Ca^2+^ modulators	Afterload
Role of Interaction	Motor allocation	Utilization of allocated motors	Output of ability (force and/or velocity)

#### 2.5.3 It is inaccurate to consider intrinsic factors independent of extrinsic factors

Whether the intrinsic resources of the capacity/resource domain are independent of extrinsic factors depends on whether the focus is on the current state or long-term adaptation. In the current state, the capacity/resource domain can be considered intrinsic and independent of acute extrinsic factors (e.g., the 3-imEFs). However, over the long term, it becomes highly dependent on extrinsic factors. For example, chronic elevation of afterload can trigger compensatory hypertrophy, increasing sarcomere numbers and enhancing myosin binding sites and Ca^2^⁺ transient capacity (Kontaridis et al., 2015). Conversely, chronic myocardial disease can lead to atrophy and scarring, reducing the available resources. Environmental factors, such as sustained thermal shifts or microgravity, can also reshape the capacity/resource domain. These long-term adaptations reflect chronic adaptability.

The adaptability domain governs both acute and chronic responses. Acute adaptability involves utilizing the capacity domain to generate appropriate F and/or V in response to a given set of 3-imEFs. Chronic adaptability drives structural and functional modifications within the capacity/resource domain to meet long-term demands. Thus, adaptability serves as the regulatory link between capacity and ability, ensuring both the proper use of resources and their long-term evolution.

Without adaptability, ability cannot be generated and capacity cannot be accessed and utilized. Therefore, MC is best conceptualized as **MC = MC_α_ ∪ MC_β_ ∪ MC_γ_**, where ∪ denotes the union of the following:

• **MC_α_** = Adaptability functional domain (regulatory mechanisms)
• **MC_β_** = Ability functional domain (emergent mechanical consequences)
• **MC_γ_** = Capacity functional domain (resources)

The union symbol is used instead of summation to avoid implying linearity, as MC is an emergent property of myocardial tissue with complex, non-linear interactions.

## 3 A conceptual, functional framework that conceptualizes MC and characterizes myocardial contraction

In this section, we present the rationales for developing a conceptual, functional framework that conceptualizes MC comprehensively from a broader perspective, establish the framework, illustrate how the framework and the concepts of emergent properties and nonlinearity improve diagnostic or therapeutic strategies, address the effectiveness of the framework, and differentiate between the capacity/resource domain and cardiac reserve, a term commonly used in clinical practice.

### 3.1 Rationales

Necessity: The complexity of MC is evident, with two common tendencies in the literature: defining MC within a purely mechanical scope and oversimplifying it with a single index or sentence. Among the works reviewed, Blinks and Jewell (1972) provided the most comprehensive analysis within the mechanical framework but ultimately concluded that a rigorous definition of MC was impossible. We align with Einstein’s insight that “no problem can be solved from the same level of consciousness that created it,” emphasizing the need for a higher-level, broader conceptualization of MC through a CAS perspective. Furthermore, myocardial contraction is characterized by MC, relaxation, and high resistance to fatigue, with MC as a key emergent property. However, current definitions mostly reflect only the ability domain, focusing on the output (F and/or V), which fails to fully characterize myocardial contraction. A more comprehensive approach must include how resources are allocated, utilized, and how ability emerges.

Feasibility: In the previous section, we reasoned out the three functional domains of MC. With a further explanation of the nonlinear relationships among these domains, the framework can be established.

Suitability: The suffixes “-ity” in capacity and “-ility” in adaptability and ability trace back to the Latin “-itas” or “-itās,” meaning the state, quality, or condition of something. This etymology underscores the suitability of “contractility” as an umbrella or comprehensive term for these three domains, each representing a distinct but interconnected aspect of MC as an active emergent property characterizing a single contraction.

### 3.2 Establishment of the MC framework

**FIGURE 2:**
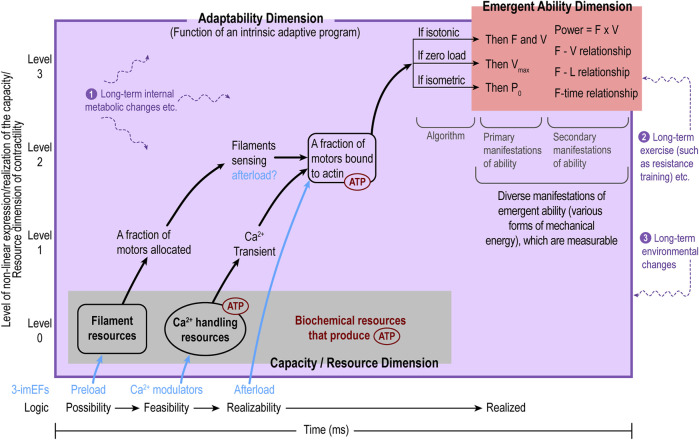
The internal functional organization of myocardial contractility (MC) follows the A-U-O pattern: regulated capacity/resource Allocation (A), regulated Utilization of allocated capacity/resource (U), and regulated final Output (O, the ability domain). On the X-axis, the A-U-O pattern reflects the possibility-feasibility-realizability temporal sequence, while the Y-axis shows the level of capacity/resource utilization/expression. The adaptability domain (purple) encompasses both the capacity/resource domain (gray) and the emergent ability domain (red) as it regulates the entire contraction process. The adaptability domain can be viewed as a complex regulatory program. So far, only one subprogram (i.e., the algorithm with three if-then scenarios in response to various afterload conditions) is inferred in Section 2.5.1 in the main text. The emergent ability domain as the functional output of a single contraction is exposed to the outside afterload and is visible and measurable, whereas the capacity/resource and adaptability domains are intrinsic and invisible. The purple texts and purple, wavy, dashed lines represent various long-term intrinsic or extrinsic factors that may cause long-term changes in all three domains of MC as a whole. From the capacity/resource domain to the ability domain, the expression processes are non-linear.

The relationships among the three functional domains of MC for a single contraction can be abstracted as a sequence: regulated capacity/resource Allocation, regulated resource Utilization, and regulated final Output (ability domain); “regulated” reflects the role of the adaptability domain throughout the process. This functional sequence is defined as the A-U-O pattern, depicted in Figure 2.

Five points should be noted. First, the MC framework naturally applies to an individual cardiomyocyte to describe cellular contractility. Second, since the three domains in the framework are shared by all cardiomyocytes, it can be extended to describe MC at the myocardial tissue level. However, third, when applying it to the tissue level, the heterogeneities among cardiomyocytes are not taken into consideration, which is a limitation of the MC framework. Another limitation is that only the outputs of the ability domain are measurable (as primary P_0_, V_max_, F, and V and secondary F-V, F-L relationships, etc.), so evaluations of the performances of the capacity/resource and adaptability domains are not possible currently. However, the MC framework can guide research and clinical practice toward this direction, as described in the next section. Fourth, MC itself can be considered a functional CAS affiliated with myocardial contraction. It is an open system, subject to corrections, modifications, and new developments because myocardial contraction is extremely complex. Finally, although MC is an active property of muscle contraction, it cannot be isolated from the passive properties of the myocardial tissue but is influenced by them.

Aboelkassem and Trayanova (2019) modeled tropomyosin dynamics using a multi-well energy landscape. Their mathematical approach offers theoretical support to our conceptual and qualitative CAS approach to MC and a valuable foundation for future quantitative modeling of our framework, particularly in understanding how Ca^2^⁺ modulators influence the functional states of the myocardium.

### 3.3 Diagnostic or therapeutic implications of the three domains

Identifying the primary and secondary changes in the three domains informs training or therapeutic strategies. For example, during acute exercise, MC is primarily enhanced through the adaptability and ability domains, while the capacity/resource domain remains unchanged. The adaptability domain regulates β-adrenergic stimulation, increasing Ca^2^⁺ transient amplitude, speed, and calcium sensitivity, which enhances resource utilization without altering the capacity of all resources. This results in a non-linear enhancement of the output of the ability domain to meet the increased metabolic demand. In contrast, ischemia primarily affects the capacity domain and possibly the adaptability domain, leading to a decline in ability. Structural damage and oxygen deprivation reduce filament availability, ATP production, and Ca^2^⁺ handling efficiency, compromising the capacity/resource domain. Simultaneously, the adaptability domain becomes dysregulated, as excessive sympathetic activation attempts to compensate but eventually results in impaired calcium handling. Despite the initial injury, functional decline in the ability domain is non-linear, as compensatory mechanisms can temporarily preserve myocardial output before functional collapse. These examples highlight the non-linear interactions among the three domains, where adaptability can optimize performance without changing the capacity of the resource domain, while disruptions in capacity can trigger disproportionate functional decline.

Clinically, this framework suggests that positive inotropic agents, commonly used to increase MC in heart failure, may worsen ischemic injury. By amplifying calcium influx and cross-bridge cycling, these agents increase oxygen demand and cellular stress, potentially accelerating damage in already compromised tissue, similar to the way driving a worn-out car engine at maximum speed risks further structural damage. This view highlights the need for interventions that preserve capacity and optimize adaptability rather than merely enhancing contraction force as follows.

During ischemia, microvascular dysfunction is a new emergent property (consequence). Even after reperfusion, microcirculatory disturbances can persist, contributing to ongoing injury and adverse remodeling (Hausenloy and Yellon, 2013). Mechanisms such as endothelial injury and nitric oxide dysregulation provide opportunities for targeted therapies, including agents that restore endothelial function or promote angiogenesis (Wang and He, 2020). Similarly, ischemia-induced metabolic shifts from aerobic to anaerobic metabolism, with lactate accumulation and ATP depletion, can impair contractility and lead to further injury. Metabolic interventions, such as glucose- and fatty acid-based therapies, have been explored as adjunctive strategies to protect ischemic myocardium (Taegtmeyer, 2004; Jaswal et al., 2011).

**FIGURE 3:**
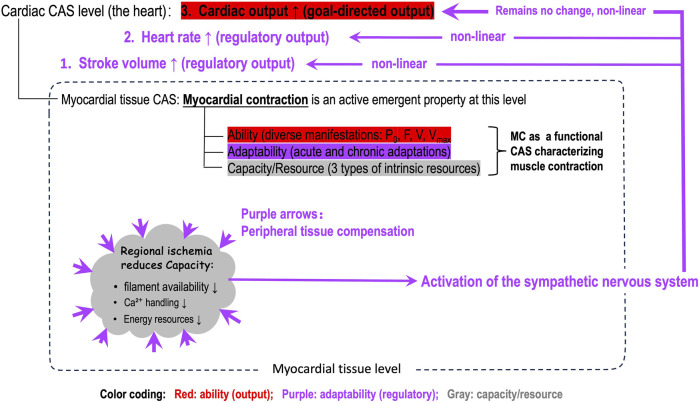
An illustration of the position of myocardial contractility (MC) in the whole-heart, the role of MC, and the nonlinear dynamics among the three functional domains (capacity/resource, adaptability, ability domains) of MC after a regional ischemia in the myocardium. Note: The nonlinear regulatory dynamics are layered and adaptability domain of MC is integrated in the higher-level regulatory mechanism.

Figure 3 further illustrates that positioning MC at the myocardial tissue level precisely defines its role: characterizing myocardial contraction comprehensively. So far, the heart- or ventricular-level parameters (stroke volume, ejection fraction, ESPVR, etc.) are confused with the tissue-level MC, which is disadvantageous for personalized and precise medicine. Figure 3 also explicitly differentiates what output of the whole heart is goal-directed and what outputs serve regulatory roles. This differentiation better illustrates the layered, non-linear dynamics between the myocardium and the levels beyond it.

### 3.4 Effectiveness of the MC framework

Within the MC framework, all issues discussed in the previous section are eliminated. As shown above, MC and cardiac performance are clearly differentiated. And the framework clarifies and extends Muir and Hamlin’s (2020) modern definition by clearly distinguishing adaptability from ability. Their description of “preload, afterload, and length-independent intrinsic” maps to the capacity/resource domain, while their “kinetically controlled, chemo-mechanical processes responsible for force and velocity” merges adaptability with ability. Our three-domain framework separates adaptability and ability, providing greater clarity and also incorporating long-term chronic adaptation.

### 3.5 Capacity domain vs. cardiac reserve

The concept of cardiac reserve is commonly used to describe the heart’s ability to increase contractile performance under stress, often linked to Ca^2^⁺ handling mechanisms and β-adrenergic stimulation at the whole-heart level. It primarily reflects a *functional* (not structural) reserve used to augment contractility in response to increased demands. At the myocardial tissue level, a similar concept can be described as myocardial reserve, which refers to the tissue’s functional capacity to enhance contractility when needed. Our capacity domain is broader, encompassing not only this functional reserve but also structural resources (e.g., the total number of myosin heads and actin binding sites) and functional resources (e.g., Ca^2^⁺ handling capacity and ATP availability). It represents the total intrinsic resources available for contraction, independent of their recruitment during a single contraction cycle.

A critical distinction lies in the nonlinear relationship between myocardial reserve (tissue level) and cardiac reserve (whole-heart level). A reduction in myocardial reserve due to localized dysfunction, such as ischemia, may not immediately lower cardiac reserve due to compensatory mechanisms like increased recruitment of healthy myocardial regions. However, once these mechanisms reach their limits, further reductions in myocardial reserve can result in a disproportionate decline in cardiac reserve. This difference emphasizes the importance of conceptualizing MC from the perspective of a complex adaptive system (CAS). Recognizing nonlinear dynamics and emergent properties is crucial for accurately assessing MC and understanding how tissue-level impairments can influence whole-heart performance.

## 4 Significance of the new conceptual developments beyond the scope of MC

The significance of several new conceptual developments is addressed progressively, moving from more specific values to broader, more impactful implications, encompassing both theoretical and practical values.

### 4.1 The definition and use of the term of 3-imEFs fill a logical gap

Grouping preload, Ca^2^⁺ modulators, and afterload as the three immediate extrinsic factors (3-imEFs) is necessary for three reasons: First, they are indeed acute extrinsic factors modulating the capacity/resource domain to generate functional ability, distinct from long-term influences. Second, this article is the first to present their sequential roles from possibility to feasibility to realizability in a complete time-series progression (Table 1), advancing the theoretical understanding of MC beyond experimental studies. Third, despite their sequential importance in completing a contraction, textbooks and literature often list only preload and afterload as extrinsic factors, while contractility remains vaguely defined as an intrinsic factor. Omitting Ca^2+^ modulators as a parallel extrinsic factor equivalent to preload and afterload reveals a logical gap. By defining them as 3-imEFs, we place Ca^2+^ modulators in their proper context, addressing this gap and completing a systematic, time-sequenced view of a single muscle contraction.

### 4.2 Non-linearity guides research and clinical practice

In the coordinates, the non-linear dynamics of a process means that any change of the framework must involve changes in the X-axis (resource flow) and/or the Y-axis (functional state); however, a change in the X-axis and/or Y-axis may not always result in a change in the overall function, unless critical thresholds are reached. For example, a membrane potential increase may only trigger an action potential if a threshold is crossed, while the oxygen dissociation curve shows that hemoglobin saturation changes minimally at high O_2_ partial pressure but shifts dramatically in the mid-range. Similarly, research and clinical practice should focus on identifying the pivotal thresholds where small changes can trigger disproportionate effects, aiding early intervention strategies. In summary, recognizing non-linearity helps maximize efficiency, achieving greater impact with minimal input—an insight with relevance beyond MC.

### 4.3 The A-U-O pattern may be a universal framework for studying biological complex systems

The A-U-O (Allocation-Utilization-Output) pattern is natural, logical, and internally consistent, making it potentially applicable beyond the MC framework. For example, in gene expression, regulated genetic resource Allocation (epigenetically selected gene expression) is followed by regulated Utilization (transcription) and then the regulated Output as structural and functional phenotypes. Similarly, the primary goal of glomerular blood flow regulation is to maintain a normal glomerular filtration rate (GFR, goal-directed Output). This is achieved by renal autoregulation, initially modulating afferent arteriole tone (Allocation and Utilization of afferent arterioles and nephrons). When these mechanisms are insufficient due to a significant drop in renal blood flow, it is possible that resource Allocation expands to include efferent arterioles to constrict (Utilization). So, it is still likely to maintain the normal GFR Output even the normal RPF cannot be maintained (Denton et al., 2000; Kuang et al., 2024).

## 5 System-level emergent properties of MC

Beyond the internal organization of the MC framework, considering MC to be a functional, tissue-level CAS, the following system-level emergent properties that influence all three domains of MC can be highlighted briefly.

### 5.1 Efficiency

The efficiency of MC (Mechanical energy produced during muscle contraction/Total energy expenditure, Gibbs and Barclay, 1998; Han et al., 2022) should be considered an emergent property because it is not just about energy expenditure (e.g., ATP use during cross-bridge cycling and Ca^2^⁺ reuptake) but an outcome of the dynamic integration of the capacity, adaptability, and ability domains.

Understanding efficiency as a system-level emergent property has several implications. It could help tailor treatments for cardiac conditions like heart failure by focusing not just on energy usage (e.g., ATP production) but also on enhancing muscle capacity and adaptability. Drugs and therapies could target multiple pathways for improved heart function. Exercise training programs could be designed to optimize muscle adaptability and ability, improving overall performance, rather than focusing solely on energy efficiency. Studying the various patterns of adaptive heterogeneity is a challenging but necessary task to deepen the understanding of cardiac performance.

### 5.2 Circadian rhythm-dependency

In mammals, all body cells, including cardiomyocytes, have intrinsic circadian clocks that regulate various cellular processes, synchronizing with the central circadian clock in the hypothalamus, which coordinates biological rhythms throughout the body (Durgan and Young, 2010; Martino and Young, 2015; Crnko et al., 2019; Lal et al., 2024). These intrinsic clocks influence cellular functions like metabolism, gene expression, epigenetic regulation, and signaling. Consequently, all domains of MC should be circadian rhythm-dependent, with contractile function varying throughout the day. Maximal isometric tension in the human heart typically peaks during the late afternoon or early evening, aligned with peak circadian gene expression (Malik et al., 2020; Lal et al., 2024). By aligning research experiments, exercise training, drug administration, and so forth with the natural circadian rhythm, it is possible to optimize outcomes, enhance efficacy, and reduce risks in cardiovascular health management.

### 5.3 History-dependency

Cardiac muscle function is history-dependent, meaning its current function is influenced by its prior activity or conditions. For example, the Bowditch effect (staircase phenomenon or frequency-dependent activation) illustrates how an increase in heart rate (or stimulation frequency) leads to a gradual enhancement of contraction strength, largely attributed to increased Ca^2+^ availability in the cardiomyocytes (Endoh, 2004; Usman et al., 2023). The Frank-Starling mechanism is another example of history-dependent myocardial function. It demonstrates that a previous increase in ventricular filling (preload) enhances subsequent contraction strength by optimizing actin-myosin overlap within the sarcomeres. The history-dependent nature of myocardial function highlights the value of progressive adaptation in exercise training and preventing overtraining and maladaptive changes in the heart. Future researchers can place an explicit focus on optimizing the ability domain of MC through optimizing the adaptability domain, which encompasses more efficient utilization of the capacity/resource domain.

### 5.4 Temperature-dependency

Cellular activities or events, such as enzyme activities, signal transduction pathways, protein folding and stability, membrane fluidity, and gene expression, are temperature-dependent. Hence, temperature changes influence all three domains of MC. Both external and internal heat affect the heart’s thermal environment (Gambassi et al., 1994; Dietrichs et al., 2019). In general, as the temperature rises, the rate of biochemical reactions increases, which can enhance the contractile performance of cardiac muscles by accelerating the rate of cross-bridge cycling between actin and myosin filaments. However, excessively high temperatures may lead to protein denaturation and impaired cardiac function. Conversely, lower temperatures generally slow down these biochemical processes, reducing the rate of contraction and the force generated (Fenton et al., 2013). This temperature-dependency is crucial for understanding the physiological limits of myocardial function under varying thermal conditions and can have significant implications for clinical settings where body temperature deviates from normal ranges (Moghadamnia et al., 2017).

### 5.5 Notes

In terms of the system-level properties of MC, the following points need to be addressed. First, due to the interconnectedness and interdependence of the functions from the molecular level to the cellular, myocardial tissue, and heart levels, these system-level properties may be shared by all levels of functions in the heart and thus may be extended to be the system-level properties of the entire heart. Second, the four properties highlighted above are not exclusive. Third, the previous two points can be exemplified using the concept of adaptability, which can be applied to all levels in the heart, manifested as adaptive changes in some signal transduction pathways, epigenetic regulation, functions of cardiomyocytes, myocardial tissues, and the entire heart. Finally, when the term “adaptability” is used, it is necessary to provide the scope or level at which it applies. The adaptability domain in the MC framework (Figure 2) applies specifically to the MC framework and is responsible for the allocation and utilization of the capacity/resource domain and production of the ability of the myocardium.

## 6 Conclusion

Myocardial contractility (MC) cannot be defined simply using a single index or sentence. Applying the concept of complex adaptive systems and based on the conceptual analysis presented here, the outcomes of this article are summarized below:

First, a three-domain framework is established at the myocardial tissue level to conceptualize MC from a broader scope, including the functionally interwoven domains of capacity/resource, adaptability, and ability. These domains interact dynamically, and their relationships follow a functional sequence of regulated resource Allocation (A), regulated resource Utilization (U), and regulated functional Output (O), referred to in this article as the A-U-O pattern.

Second, the three-domain MC framework clearly distinguishes MC from whole-heart functional parameters such as stroke volume, ejection fraction, ESPVR, and so forth and eliminates all issues with the current definitions of MC addressed in this article.

Third, the system-level behaviors of MC are highlighted, including the efficiency of muscle contraction, circadian rhythm-dependency, history-dependency, and temperature-dependency; these concepts are of value to guide research on cardiac contraction.

Fourth, the three-domain MC framework suggests the need to optimize resource utilization rather than focus solely on maximizing contractile output, potentially improving strategies for managing heart failure and personalizing medicine.

Fifth, the significance of the A-U-O pattern extends beyond our three-domain framework of MC and can serve as a universal pattern for studying biological complex systems.

Sixth, the significance of non-linearity also extends beyond our three-domain framework and is important to guide biomedical research and clinical practice in identifying thresholds in a dynamic process, optimizing therapeutic effect or preventing deterioration of a disease.

This three-domain framework is an initial attempt to redefine MC systematically. It remains open for constructive critique, refinement, and expansion as new experimental and clinical insights emerge. Future research will involve adopting the methodology in Aboelkassem and Trayanova (2019) and mathematical modeling of the three-domain MC framework.

## Data Availability

The original contributions presented in the study are included in the article/supplementary material, further inquiries can be directed to the corresponding author.
